# Protective Effect of Bilberry Anthocyanin Extracts on Dextran Sulfate Sodium-Induced Intestinal Damage in *Drosophila melanogaster*

**DOI:** 10.3390/nu14142875

**Published:** 2022-07-13

**Authors:** Guocai Zhang, Yunyao Gu, Xianjun Dai

**Affiliations:** College of Life Sciences, China Jiliang University, Hangzhou 310018, China; zhanggc1996@126.com (G.Z.); yunyaogu@126.com (Y.G.)

**Keywords:** bilberry anthocyanin, *Drosophila melanogaster*, inflammatory bowel disease, antioxidant, intestinal microflora

## Abstract

Inflammatory bowel disease (IBD) is a chronic recurrent disease that can be controlled by various natural extracts. Anthocyanins (ANCs) from bilberry have significant antioxidant capacity and are widely used as food colors and antioxidants. In this study, we investigated the protective effects of bilberry anthocyanin extracts (BANCs) against dextran sulphate sodium (DSS)-induced intestinal inflammation in a *Drosophila melanogaster* (*D. melanogaster*) model, and the effects on the lifespan, antioxidant capacity, intestinal characteristics, and microbiome and gene expression profiles were analyzed to elucidate the underlying biological mechanisms. In DSS-induced normal and axenic *D. melanogaster*, BANCs significantly increased the survival rate, maintained the intestinal morphology and integrity, and reduced the number of dead intestinal epithelial cells and the ROS level of these cells. BANC supplementation had no significant effect on the intestinal microflora of DSS-induced *D. melanogaster*, as demonstrated by a 16S rDNA analysis, but improved the antioxidant capacity by activating the relative gene expression of NRF2 signaling pathways in the intestine of *D. melanogaster* with DSS-induced inflammation. Therefore, the results demonstrate that BANCs effectively alleviate intestinal inflammatory injury induced by DSS and improve the antioxidant capacity of *D. melanogaster* by modulating NRF2 signaling pathways, and could thus promote the application of BANCs as functional foods.

## 1. Introduction

Inflammatory bowel disease (IBD) is a chronic recurrent disease that is known to cause life-long recurrence of abnormal inflammation in the intestinal tissue. Disruption of the epithelial barrier represents the initiating step of intestinal inflammation. Epithelial cells, which provide structural barrier components from the secreting cells, are sensors of the external environment, and emit signals that regulate the underlying innate and adaptive immunity [1]. Recent data from animal models show that intestinal inflammation can be initiated by molecular defects restricted to the epithelium in the presence of a normal microbial flora and normal underlying innate and adaptive immunity [2]. At present, IBD is a global public health problem, and untreated IBD is likely to cause complications, such as peritonitis, or to progress to colorectal cancer [3]. However, despite an understanding of the various causes of IBD, the currently available therapies are limited, and no drugs are currently available for completely curing IBD [4]. Therefore, various natural extracts have been reported as preventive drugs against the disease.

Anthocyanins (ANCs) are flavonoid compounds with structures consisting of two aromatic rings separated by a heterocyclic ring with an oxygen cation. ANCs have a broad spectrum of biological activities, including antioxidant and anti-inflammatory activities, vision improvement, and protection of vascular function [5,6,7,8] and anticarcinogenic properties, including an impact on autophagy regulation in tumor development [9,10]. At present, ANCs are widely used for preserving food, cosmetics, healthcare, and other applications, and have high development and utilization value [11]. Some studies have shown that ANCs can protect the intestine by reducing tumor necrosis factor-α, modulating oxidative stress, promoting intestinal barrier function, and regulating the gut microbiota [12,13,14]. Bilberry (*Vaccinium myrtillus*) is a perennial bush plant whose blue and black fruits are a result of a combination of anthocyanidins (delphinidin, cyanidin, petunidin, malvidin, and peonidin) and sugar components (glucose, galactose, and arabinose) [15]. In traditional medicine, bilberry is used to treat varicose veins, venous insufficiency, fatigue, diarrhea, and gastrointestinal complaints [16]. Hence, bilberry fruits represent an attractive food product with potential value in the prevention and/or treatment of different conditions associated with inflammation, dyslipidemia, hyperglycemia or increased oxidative stress, cardiovascular disease (CVD), cancer, diabetes, and dementia and other age-related diseases [17]. However, only very limited studies explain whether the ameliorating effect of bilberry anthocyanin extracts against inflammatory injury of the intestine is related to improving the health of intestinal microflora or through the underlying signaling pathway. In this study, we evaluated the effects of BANCs on the lifespan, intestinal characteristics, and intestinal microflora of dextran sulfate sodium (DSS)-treated *D. melanogaster*, and explored the mechanism through which BANCs alleviate intestinal inflammatory injury.

## 2. Materials and Methods

### 2.1. Materials

BANCs were prepared by the extraction with ethanol after enzymatic hydrolysis by Zhejiang Huiyuan Pharmaceutical Co., Ltd. (Huzhou, China). The relative ingredient values were analyzed using HPLC–MS methods by Qingdao Kechuang Quality Testing Co., Ltd. (Qingdao, China). Five ingredients were determined in the extracts, and these included delphinidin 3-*O*-glucoside (14,178.80 μg/g), cyanidin 3-*O*-arabinoside (100.69 μg/g), peonidin 3-*O*-glucoside (313.82 μg/g), cyanidin (1877.14 μg/g), and pelargonidin (25.97 μg/g). All other chemicals, such as DSS (M.W. 500000, Shanghai Macklin Biochemical Co., Ltd., Shanghai, China), 7-aminoactinomycin (7-AAD), 4A Biotech Co., Ltd., (Beijing, China), 4′,6-diamidino-2-phenylindole (DAPI, Beijing Solarbio Science & Technology Co., Ltd., Beijing, China), dihydroethidium (DHE, 4A Biotech Co., Ltd., Beijing, China), erioglaucine disodium salt (Sigma Co., Ltd., St. Louis, MO, USA), and MDA kit and protein standards (Nanjing Jiancheng Bioengineering Co., Ltd., Nanjing, China), were of analytical grade.

### 2.2. Fly Stock, Culture, and Treatment

The Cation S *D. melanogaster* line was obtained from the *Drosophila* Stock Center at the Shanghai Academy of Life Sciences, Chinese Academy of Sciences. The emerged flies were raised on basal medium (20.4 g of corn flour, 15.6 g of sucrose, 1.68 g of yeast powder, 1.8 g of agar powder, 1.2 mL of propionic acid, and 190 mL of distilled water) at 25 °C under a relative humidity (55%) and a 12-h light/12-h dark cycle. After 3 days, female *D. melanogaster* were separated by slight anesthesia with CO_2_ and transferred to a vial with filter paper that contained 0.1 mg/mL BANCs in 5% sucrose (CA, BANC control group), 7% DSS and 0.1 mg/mL BANCs in 5% sucrose (DA, BANCs + inflamed group), or 7% DSS in 5% sucrose (D, inflamed group), or only filter paper saturated in 5% sucrose (C, blank control group). The filter paper was changed every 12 h.

To obtain axenic *D. melanogaster*, the eggs were collected in grape-juice-yeast medium (750 mL of ddH_2_O, 250 mL of grape juice, 30 g of agar, and 12 g of sucrose), to the surface of which, several drops of yeast paste were added, placed inside 1% active chlorine for sterilization for 5 min, and then repetitively rinsed with 70% ethanol for 1 min and sterile H_2_O for 1 min. The embryos were then transferred to autoclaved basal medium in an incubator (25 °C, 55% humidity). Newly emerged *D. melanogaster* were randomly divided into the C, CA, D, and DA groups under slight anesthesia with CO_2_ gas under sterile conditions.

### 2.3. Lifespan Assays

Twenty female flies in each tube (10 tubes in each group, 200 flies in each group), were transferred into a culture tube with filter paper soaked in the corresponding medium. The natural time at which each *D. melanogaster* individual in each tube died was recorded until all *D. melanogaster* died, and the mean life, maximum life, and half-to-death life were calculated for each experimental group. Axenic *D. melanogaster* were maintained in a sterile incubator throughout their lifespan.

### 2.4. Smurf Assays

Erioglaucine disodium salt is a blue dye that can spread throughout the fly as a result of intestinal leakage [18]. Because DSS can destroy the colonic epithelium of *Drosophila* [19], we examined the intestinal permeability of *D. melanogaster* treated with DSS in the presence or absence of BANCs. The intestinal permeability was measured using the Smurf test, which was based on monitoring the presence of unabsorbed blue dye outside the digestive tract. Female flies were placed in a tube with filter paper saturated in 5% sucrose solution for 72 h, starved for 1 h, and then cultured in another tube with filter paper saturated in 5% sucrose solution containing 2.5% erioglaucine disodium salt for 12 h. The flies were then observed under a microscope for the evaluation of Smurf leakage, and the leakage rate was calculated according to the percentage of blue fruit flies within the total fruit fly population.

### 2.5. Intestinal Morphology Assays

In *D. melanogaster,* gut-damaging agents induce morphological shortening of the midgut [19]. After 72 h of culture, female *D. melanogaster* were starved for 1 h, and the whole intestine was dissected and extracted in ice-cold PBS solution. The intestinal tract was fixed with 4% paraformaldehyde for 20 min and then washed three times with PBS. After the addition of 70% glycerol, the tract was placed under an electron microscope for observation and photography, and the intestinal length was measured using ImageJ software(×64)1.8.0 from National Institutes of Health (Bethesda, Rockville, MD, USA).

### 2.6. 7-AAD Assays

7-AAD can enter dead cells and bind to DNA, and emits red fluorescence at an excitation wavelength of 546 nm [20]. To explore intestinal injury induced by DSS, the dissected guts of *D. melanogaster* were stained with 7-AAD. Briefly, the dissected intestines were combined with 7-AAD at room temperature under dark conditions for 30 min and then washed three times with PBS. The intestines were fixed with 4% paraformaldehyde for 30 min, washed three times with PBS, incubated with DAPI for 5 min, and washed three times with PBS. The tablets were then sealed with 70% glycerol and observed and photographed with a fluorescence microscope. The number of dead intestinal epithelial cells was determined based on the fluorescence intensity of 7-AAD using ImageJ software.

### 2.7. Intestinal Epithelial Cell ROS Assays

Dihydroethidium (DHE) can freely enter cells through living cell membranes, and is oxidized by intracellular ROS to form ethyl oxide. Ethyl oxide can be incorporated into chromosomal DNA to produce red fluorescence. Thus, the ROS content in cells can be determined according to the production of red fluorescence in living cells [21]. The dissected intestines were combined with DHE for 30 min at room temperature and then washed three times with PBS. The intestines were fixed with 4% paraformaldehyde for 30 min, washed three times with PBS, incubated with DAPI for 5 min, washed three times with PBS, and sealed with 70% glycerol. The specimens were then observed and photographed with a fluorescence microscope. The ROS content in intestinal epithelial cells was determined based on the fluorescence intensity of DHE, measured using ImageJ software.

### 2.8. RNA Isolation and Real-Time Quantitative PCR

The guts of *D. melanogaster* were dissected and homogenized, and total RNA was isolated using TRIzol reagent (RNAiso Plus) following the manufacturer’s instructions. The PCR program was as follows: an initial temperature of 95 °C for 30 s, followed by 39 thermal cycles of 94 °C for 5 s and 60 °C for 30 s. The primers were designed and synthesized by Wcgene Biotech, Shanghai, China. The Rp49 gene was used as the reference gene, and the calculations were performed using the 2^−ΔΔCt^ method. The primer sequences are shown in Table 1.

### 2.9. Antioxidant Capacity Assays

Fifty fruit flies from each group were collected, fasted for 2 h, and weighed. In an ice bath, the flies were mixed with physiological saline solution at a ratio of 1:9 (fruit fly weight: physiological saline). The mixture was then homogenized with a glass homogenizer and centrifuged at 4 °C and 560 g/min for 15 min. The protein and malondialdehyde (MDA) contents were measured using kits according to the manufacturer’s instructions.

### 2.10. Intestinal Microbiome Assays

16S rDNA was used to analyze the *D. melanogaster* intestinal microbial composition. Briefly, the midgut was dissected as described above, and DNA from different midgut samples was extracted using the E.Z.N.A.^®^ Stool DNA Kit (D4015, Omega, Inc., Norcross, GA, USA) according to the manufacturer’s instructions. The total DNA was eluted in 50 μL of elution buffer and stored at −80 °C until measurement. The results of the 16S rDNA sequencing analysis were obtained from LC-Bio Technology Co., Ltd., Hangzhou, Zhejiang Province, China. Briefly, amplicon pools were prepared for sequencing, and the size and quantity of the amplicon library were assessed with an Agilent 2100 Bioanalyzer (Agilent, Santa Clara, CA, USA) and the Library Quantification Kit for Illumina (Kapa Biosciences, Woburn, MA, USA), respectively. The libraries were sequenced using the Illumina NovaSeq PE250 platform. The alpha diversity (α-diversity) and beta diversity (β-diversity) were analyzed with QIIME2, and figures were drawn using R language (v3.5.2, R Core Team, Vienna, Austria).

### 2.11. Statistical Analysis

All experiments were performed with at least three replicates. The differences were analyzed for statistical significance by the two-tailed unpaired *t* test using GraphPad Prism 6 (Version No. 6.01, GraphPad Software, La Jolla, CA, USA). All data are expressed as the means ± standard deviations. *p* < 0.05 was considered to indicate statistical significance.

## 3. Results

### 3.1. Effects of BANCs on the Lifespans of Normal and Axenic D. melanogaster

**Figure 1:**
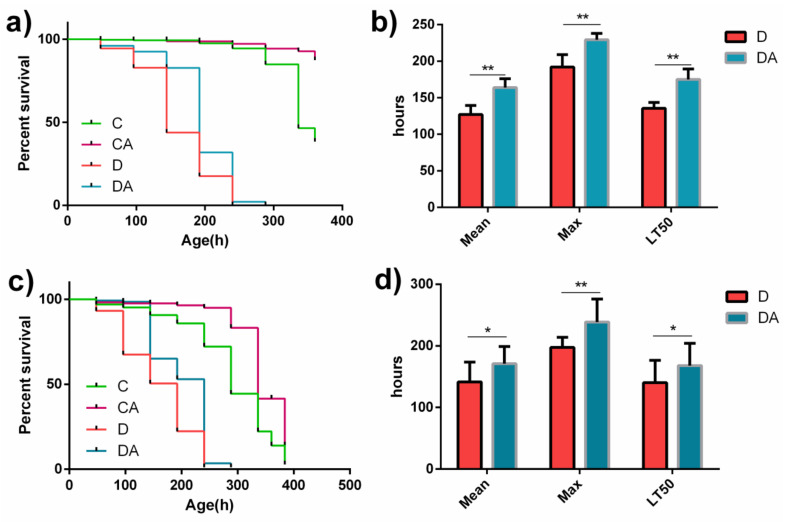
Effects of the dietary inclusion of BANCs on the lifespans of female *D. melanogaster* with DSS-induced inflammation. C, CA, D, and DA represent the blank control group, BANC control group, inflamed group, and BANCs + inflamed group, respectively. Normal (**a**) or axenic (**c**) female *D. melanogaster* were fed 5% sucrose (C), 5% sucrose containing 0.1 mg/mL BANCs (CA), 5% sucrose containing 7% DSS (D), or 5% sucrose containing 7% DSS and 0.1 mg/mL BANCs (DA). The mean, maximum (max), and median lethal time (LT50) of normal (**b**) and axenic (**d**) female *D. melanogaster* in the D and DA groups were measured. Data from triplicate repetitions are expressed as the means ± SEMs. Statistical significance: * *p* < 0.05, ** *p* < 0.01.

As shown in Figure 1a,c, the lifespans of both normal and axenic fruit flies in the BANC control group were longer than those of the corresponding flies in the blank control group, and the BANCs + inflamed group had a longer lifespan than the inflamed group. As illustrated in Figure 1b,d, BANCs significantly increased the mean lifespan (Mean), maximum lifespan (Max), and median lethal time (LT50) of normal and axenic *D. melanogaster* with DSS-induced inflammation. The mean lifespans (Mean) of the normal and axenic fruit flies in the BANCs + inflamed group were 29% (*p* < 0.01) and 20% (*p* < 0.05) longer than those of the corresponding flies in the inflamed group. The normal and axenic fruit flies in the BANCs + inflamed group had 19% (*p* < 0.01) and 21% (*p* < 0.01) longer maximum lifespans (Max) than those in the inflamed group, respectively. In addition, the median lethal times (LT50) of the normal and axenic fruit flies in the BANCs + inflamed group were 29% (*p* < 0.01) and 20% (*p* < 0.05) longer than those of the flies in the inflamed group, respectively. The above-described results show that BANCs could decrease the effect of DSS-induced inflammatory injury on the lifespans of normal and axenic *D. melanogaster*.

### 3.2. Effects of BANCs on the Intestinal Injury Index of Normal and Axenic D. melanogaster

#### 3.2.1. Effect of BANCs on the Intestine Length in Normal and Axenic DSS-Treated Flies

**Figure 2:**
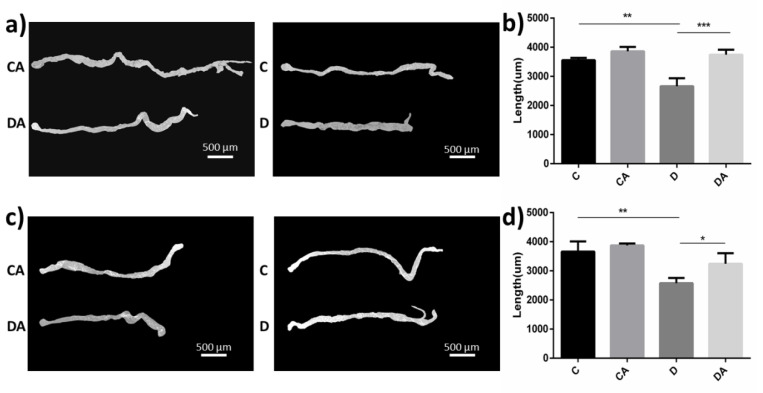
Effects of BANCs on the intestinal morphology of female *D. melanogaster* with DSS-induced inflammation. C, CA, D, and DA represent the blank control group, BANC control group, inflamed group, and BANCs + inflamed group, respectively. Intestinal morphology of normal (**a**) and axenic (**c**) female *D. melanogaster* in the four groups. The intestinal length of normal (**b**) and axenic (**d**) female *D. melanogaster* was measured using ImageJ software. Data from triplicate repetitions are expressed as the means ± SEMs. Statistical significance: * *p* < 0.05, ** *p* < 0.01, *** *p* < 0.001.

In this study, we found that DSS-induced inflammation had a significant impact on the intestinal morphology of normal and axenic flies (Figure 2a,c). The intestinal lengths of normal and axenic flies treated with DSS were reduced by 25% (*p* < 0.01) and 30% (*p* < 0.01) compared with those of the flies in the blank control group, respectively. The intestinal lengths of normal and axenic flies that were simultaneously fed DSS and BANCs (BANCs + inflamed group) were not significantly different from those of the blank control group. In addition, no significant difference in the intestinal length of normal and axenic flies was found between the blank control group and the BANCs + inflamed group (Figure 2b,d). The results indicate that BANCs can effectively protect the changes in the intestinal morphology of fruit flies resulting from DSS-induced damage.

#### 3.2.2. Effects of BANCs on the Intestinal Integrity of Normal and Sterile *Drosophila* with DSS-Induced Inflammation

**Figure 3:**
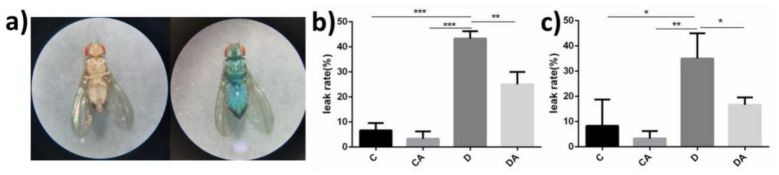
Effects of BANCs on the intestinal integrity of female *D. melanogaster* with DSS-induced inflammation. C, CA, D, and DA represent the blank control group, BANC control group, inflamed group, and BANCs + inflamed group, respectively. A blue body represents intestinal leakage in the flies ((**a**), **right**). Percentages of intestinal leakage in normal flies (**b**) and axenic flies (**c**). Data from triplicate repetitions are expressed as the means ± SEMs. Statistical significance: * *p* < 0.05, ** *p* < 0.01, *** *p* < 0.001.

After feeding, the entire body of leaky flies turned blue (Figure 3a). We found that intestinal leakage occurred in very few flies of the blank control group, whereas significant intestinal leakage occurred after DSS induction. However, no significant difference was found between normal and axenic flies. As shown in Figure 3b,c, the intestinal leakage rates of normal and axenic flies in the inflamed group were higher, reaching 43% and 35%, respectively, and the addition of BANCs reduced the rates of intestinal leakage to 25% and 17%. However, no significant difference was detected between the blank control group and the BANC control group. DSS-induced inflammation led to significant intestinal leakage, and a significant difference was found between the inflamed group and the BANCs + inflamed group (*p* < 0.01). These findings demonstrate that BANCs protect the intestinal integrity to some extent.

#### 3.2.3. Protective Effect of BANCs on Epithelial Cells in the DSS-Induced Inflamed Intestines of Normal and Axenic *D. melanogaster*

**Figure 4:**
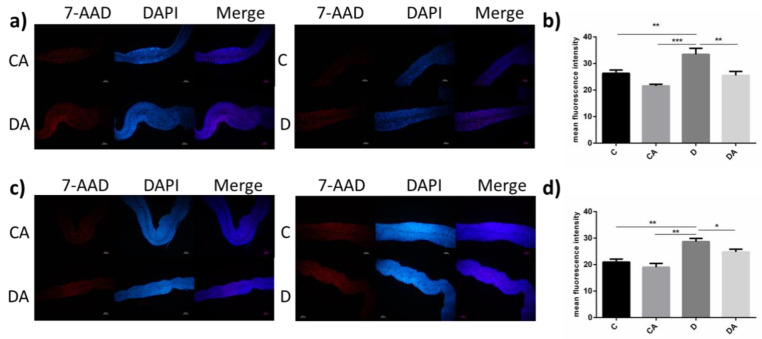
Effect of BANCs on the mortality of intestinal epithelial cells in DSS-treated female *D. melanogaster*. C, CA, D, and DA represent the blank control group, BANC control group, inflamed group, and BANCs + inflamed group, respectively. The midguts of normal (**a**,**b**) and axenic (**c**,**d**) female *D. melanogaster* in each group were stained for 7-AAD (red) and DAPI (blue). Scale bar: 50 μm. Data from triplicate repetitions are expressed as the means ± SEMs. Statistical significance: * *p* < 0.05, ** *p* < 0.01, *** *p* < 0.001.

As shown in Figure 4a,c, the number of dead intestinal epithelial cells was significantly higher among DSS-fed flies than in the blank control group, as demonstrated by the fluorescence intensity. BANCs protected the flies from DSS-induced intestinal inflammation. The analysis of dead cells based on the fluorescence intensity (Figure 4b,d) revealed significant differences in normal and axenic *D. melanogaster* between the inflamed group and BANCs + inflamed group. The mean fluorescence intensities of normal and axenic flies fed both DSS and BANCs (BANCs + inflamed group) did not significantly differ from those of the flies in the blank control group; however, the mean fluorescence intensities of normal flies fed BANCs (BANC control group) were 18% (*p* < 0.01) lower than those of the flies in the blank control group. Therefore, BANCs could reduce the injury caused by DSS-triggered inflammation in intestinal epithelial cells.

#### 3.2.4. Effects of BANCs on the ROS Content in Epithelial Cells of *D. melanogaster*

**Figure 5:**
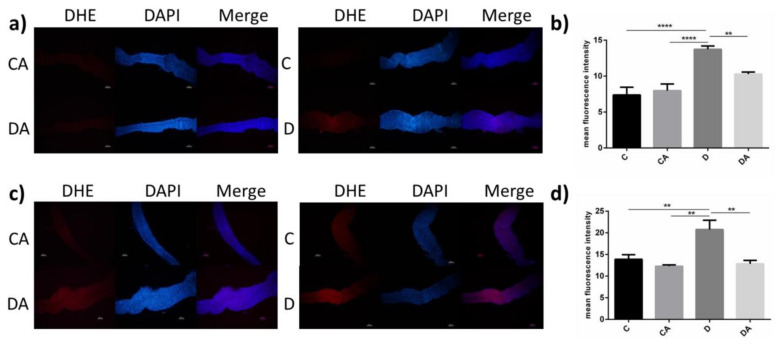
Effects of BANCs on the ROS content of intestinal epithelial cells from female *D. melanogaster* with DSS-induced inflammation. C, CA, D, and DA represent the blank control group, BANC control group, inflamed group, and BANCs + inflamed group, respectively. The midguts of normal (**a**,**b**) and axenic (**c**,**d**) female *D. melanogaster* from the four groups were stained for DHE (red) and DAPI (blue). Scale bar: 50 μm. Data from triplicate repetitions are expressed as the means ± SEMs. Statistical significance: ** *p* < 0.01, **** *p* < 0.0001.

DSS significantly increased the ROS levels in intestinal epithelial cells, whereas dietary supplementation with BANCs significantly reduced the ROS levels in intestinal epithelial cells of normal and axenic *D. melanogaster* (Figure 5a,c). A fluorescence intensity analysis (Figure 5b,d) revealed significant differences in normal and axenic *D. melanogaster* between the inflamed group and the BANCs + inflamed group. The mean fluorescence intensities of normal and axenic flies fed DSS (inflamed group) was 86% (*p* < 0.001) and 62% (*p* < 0.01) higher than those of flies in the black control group, respectively. However, the mean fluorescence intensities of normal and axenic flies fed both DSS and BANCs (BANCs + inflamed group) were 25% (*p* < 0.01) and 38% (*p* < 0.05) lower than those of flies fed only DSS (inflamed group), and no significant differences in normal and axenic flies were detected between the blank control group and the BANCs + inflamed group.

### 3.3. Effects of BANCs on Intestinal Microflora of Normal D. melanogaster

**Figure 6:**
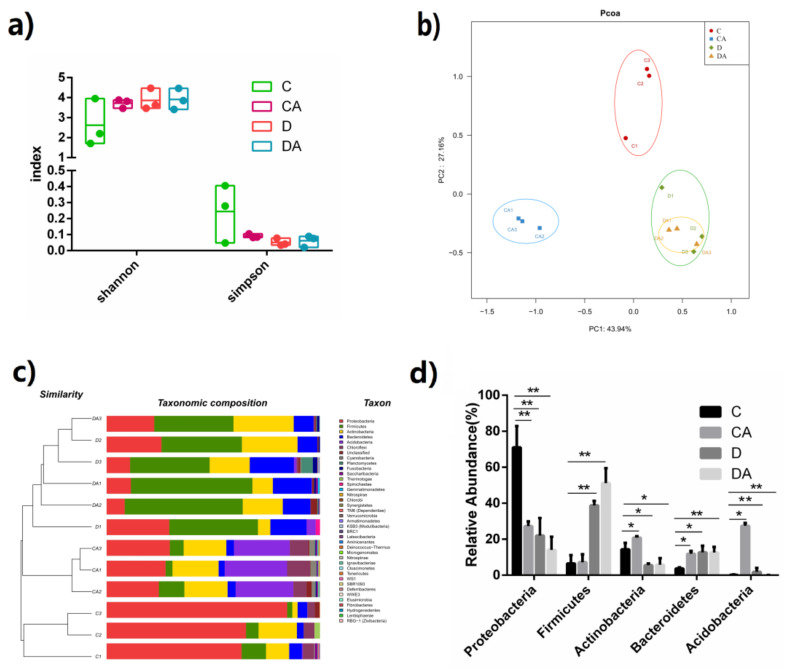
Effects of BANCs on the DSS-induced female *D. melanogaster* intestinal microbiota. C, CA, D, and DA represent the blank control group, BANC control group, inflamed group, and BANCs + inflamed group, respectively. (**a**) Effect of BANCs on the α-diversity (Shannon and Simpson index) of the midgut microbiota of DSS-exposed *D. melanogaster*. The *p* value of all the groups was obtained by the *Kruskal–Wallis* test. (**b**) Effect of BANCs on the β-diversity of the midgut microbiota of DSS-exposed *D. melanogaster* and PCoA based on weighted UniFrac distance. (**c**) Bacterial taxonomic profiling of the four groups at the phylum level (*n* = 3). (**d**) Relative abundances of five intestinal microorganisms in DSS-induced *D. melanogaster* at the phylum level. The results are presented as the means ± SEMs (*n* = 3); statistical comparisons were performed with *t* tests. Statistical significance: * *p* < 0.05, ** *p* < 0.01.

The intestinal microbiota is an important factor in the regulation of intestinal homeostasis. The Shannon and Simpson indices indicated the α-diversity of the intestinal microflora, as shown in Figure 6a, and no significant differences between the two indices of intestinal microflora abundance were found for the flies in the four groups. As demonstrated by a principal coordinate analysis (PCoA), the β-diversity of intestinal microflora revealed a similarity in the microbiota composition. The results (Figure 6b) indicated that PCoA1 and PCoA2 accounted for 43.94% and 27.16% of the overall difference in the microflora structure, respectively, and in the absence of DSS induction, the addition of BANCs to the diet of *D. melanogaster* significantly improved the β-diversity of intestinal microorganisms. However, after DSS treatment, BANCs did not significantly affect the composition of the intestinal microflora of *D. melanogaster* (Figure 6b). In Figure 6c, the relative abundances of dominant microbial species are shown at the phylum level, and the intake of BANCs significantly decreased the abundances of *Proteobacteria* from 71.07% (C group) to 27.27%, and increased the abundances of *Acidobacteria* from 0.22% (C group) to 27.25%. As illustrated in Figure 6d, the five main microorganisms in the fly intestine at the phylum level were *Proteobacteria*, *Firmicutes*, *Actinobacteria*, *Bacteroidetes*, and *Acidobacteria*, and their relative abundances in *D. melanogaster* did not indicate a significant difference between the inflamed and BANCs + inflamed groups (Figure 6d), but significant differences were found among the blank control, inflamed, and BANCs + inflamed groups.

### 3.4. Effects of BANCs on the NRF2 Signaling Pathway and Antioxidant Capacity of Normal Female D. melanogaster Exposed to DSS

**Figure 7:**
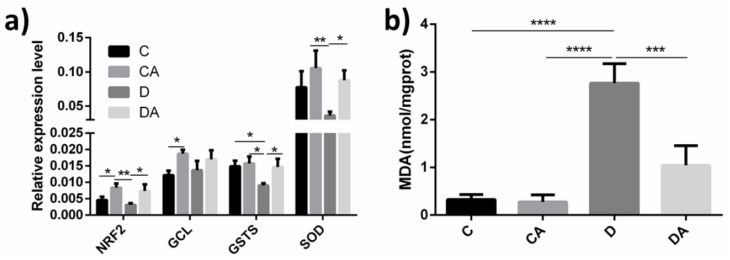
Effects of BANCs on the NRF2 signaling pathway and MDA content in DSS-treated normal female *D. melanogaster*. C, CA, D, and DA represent the blank control group, BANC control group, inflamed group, and BANC inflamed group, respectively. (**a**) Effects of BANCs on the expression levels of NRF2 signaling pathway genes. (**b**) Effects of BANCs on the MDA content. The mRNA expression levels of genes in the *D. melanogaster* guts were measured by RT–qPCR. The relative gene expression levels were normalized to Rp49. Data from triplicate repetitions are expressed as the means ± SEMs. Statistical significance: * *p* < 0.05, ** *p* < 0.01, *** *p* < 0.001, **** *p* < 0.0001.

To further explore the protective mechanism of BANCs on DSS-induced inflammation in *D. melanogaster*, the expression levels of relevant genes were analyzed by RT–qPCR. We selected and detected four genes (NRF2, GCL, GSTS, and SOD) belonging to the NRF2 signaling pathway and associated with antioxidant activities, and the results are shown in Figure 7a. In the absence of DSS treatment, BANCs could significantly increase the expression levels of NRF2 and GCL compared with the levels found in the blank control group. Exposure to DSS decreased the NRF2 and GSTS expression levels in the *D. melanogaster* gut, but the analysis of *D. melanogaster* exposed to DSS revealed that the NRF2, GSTS, and SOD gene expression levels in the BANCs + inflamed group were significantly increased to 2.35-fold (*p* < 0.05), 1.63-fold (*p* < 0.05), and 2.42-fold (*p* < 0.05) compared with those in the inflamed group, respectively. Additionally, as shown in Figure 7b, DSS exposure significantly increased the MDA content in female fruit flies compared with that in the blank control group. However, the MDA content of the BANC inflamed group was decreased by 164% compared with that of the inflamed group (*p* < 0.001). The results showed that BANCs could enhance the relative expression levels of NRF2 signaling pathway genes and reduce the MDA content in DSS-treated *D.* *melanogaster*.

## 4. Discussion

Inflammation is an important defense response process that usually causes damage to the body and affects growth and development [22,23]. Long-term intestinal inflammation may even lead to intestinal cancer and aging of the body [24,25,26]. Therefore, preventing or alleviating IBD is of great significance to the health of the body.

In this experiment, the antioxidant function of BANCs effectively alleviated inflammatory injury in the intestine, which was consistent with a previous study [27]. Compared with the flies in the inflamed group, the flies in the BANCs + inflamed group had longer intestines (similar length to that of the blank control group), improved intestinal integrity, and reduced epithelial cell mortality and intracellular ROS levels (Figure 2, Figure 3, Figure 4 and Figure 5). Further intestinal microflora analysis showed that BANCs could change the β-diversity (Figure 6b); reduce the Proteobacteria, Actinobacteria, and Acidobacteria abundances; and increase the Firmicutes and Bacteroidetes abundances (control group vs. BANC control group and BANCs + inflamed group). However, whether the protection of the intestine was dependent on a direct effect of BANCs or the BANC-mediated regulation of the intestinal microflora remains unclear, and an exploration of this question could identify many protective effects on animal intestines, such as those mediated by Lactobacillus, Bifidobacterium [28], and Saccharomyces [29]. It has been reported that anthocyanin feeding could cause changes in the intestinal microflora and attenuate inflammation [30,31]. However, the resulting alleviation of inflammation appeared to be consistent in normal and axenic flies (Figure 1, Figure 2, Figure 3, Figure 4 and Figure 5), which showed that the intestinal microflora was not involved in alleviating intestinal inflammation. The exposure of *D. melanogaster* to DSS produced a similar microbiota regardless of the inclusion of BANCs in the diet, as demonstrated by comparisons of the β-diversity (Figure 6b) and abundances of the five main microorganisms (Figure 6d) between the inflamed and BANCs + inflamed groups. Therefore, BANCs and DSS induction could change the abundances of Proteobacteria, Firmicutes, Actinobacteria, Bacteroidetes, and Acidobacteria (Figure 6d), but in the inflamed intestine, the addition of BANCs had no significant effect on these bacteria. Based on the above-described results, we speculated that BANCs could alleviate intestinal inflammatory injury in *D. melanogaster* not by regulating the intestinal microflora, but by directly acting on inflamed intestinal cells. The apparent lack of involvement of the gut microbiota in *D. melanogaster* responses to DSS was inconsistent with findings in other models [32,33], and the difference may be related to the animal model; as mentioned in the literature [32], two species, *Duncaniella muricolitica* and *Alistipes okayasuensis*, associated with worse disease outcome after DSS treatment are common, but not ubiquitous in mice, which may confound experimental results.

The intestinal inflammation occurs locally with different cell behaviors, such as proliferation, apoptosis, necrosis, and activation, and is affected by the cellular signal pathways and its downstream gene expression. The cellular pathways of regulating inflammation include NF-κB, Erk1/2, PI3K/Akt, p38, etc., among which, antioxidant signaling pathway is one of the most important regulatory pathways. In view of the above-mentioned results, we sought to determine whether BANCs directly regulate the antioxidant pathway. NRF2, as a key gene of the antioxidant signaling pathway, is a critical transcription factor that regulates target genes involved in almost every facet of metabolism, ranging from maintaining the redox balance to ensuring proper protein quality control [32,34]. The analysis of antioxidant genes in the *D. melanogaster* intestine showed that the dietary supplementation of BANCs significantly increased the relative expression levels of genes, including NRF2, GSTS, and SOD, in DSS-induced *D. melanogaster*, as observed through a comparison of the blank control and BANC control groups, but DSS induction could decrease the values of the blank control and inflamed groups (Figure 7a). The addition of BANCs to the DSS-induced flies increased the NRF2, GSTS, and SOD expression levels compared with those in the inflamed group. The above-described results confirmed the effect of BANCs in modulating the antioxidant cell pathway, which was consistent with the results reported by Jaiswal et al. [35]. The expression of genes in the NRF2 signaling pathways had a significant direct consequence on the marker levels of redox production, such as ROS and MDA (Figure 5 and Figure 7b). As shown in Figure 5a,c, BANC addition significantly reduced the ROS levels in the intestinal epithelial cells of normal and axenic *D.* *melanogaster*, which was consistent with the results reported by Zhao et al. [36]. Broadly, ROS refers to all physiologically relevant chemical species capable of reacting with macromolecules and altering their structure directly or indirectly, and includes free radicals and molecules capable of producing radicals or that are oxidizing agents themselves [37]. MDA, as the product of lipid peroxidation that causes disruption of cell membranes, is a reliable indicator of oxidative stress. ROS are the main cause of lipid peroxidation to produce MDA by destroying the lipid bilayer structure of the cell membrane [38]. An examination of the MDA content indicated that a decrease in ROS could lead to MDA reduction in inflammatory intestinal tracts fed BANCs (Figure 7b), which strongly indicated that the intake of BANCs could alleviate intestinal injury by activating antioxidant-related signaling pathways in the inflammatory intestinal tract.

## 5. Conclusions

In conclusion, BANCs alleviated intestinal injury induced by DSS by decreasing the ROS and MDA levels. The underlying mechanism could be that BANCs directly act on the NRF2 signaling pathway of intestinal cells and enhance the antioxidant capacity of *D.* *melanogaster*, but how BANCs regulate the NRF2 signaling pathway needs to be extensively studied in the future.

## Figures and Tables

**Table 1:** RT–qPCR primers.

Gene Name	Sequence (5′–3′)	Annealing Temperature	Gene Accession Number
*NRF2*	F: AGCTTCTGTCGCATGGTTGA R: AGCCGTTGCTAACATGTCCA	60 °C	NM_170055.2
*GCL*	F: GACACCGATACGCATTGCAC R: CTCACCACGGAATCCTGCTT	60 °C	NM_001298073.1
*GSTS*	F: CAGACCGTCAAGGACAACGA R: TCGCGCTTGACCATGTAGTT	60 °C	NM_166216.2
*SOD*	F: ACCGACTCCAAGATTACGCTCT R: GTTGCCCGTTGACTTGCTC	60 °C	NM_057387.5
*Rp49*	F:AGGGTATCGACAACAGAGTG R:CACCAGGAACTTCTTGAATC	60 °C	NM_079843.4

## Data Availability

Data is contained within the article.
